# Energy-Efficient Routing Control Algorithm in Large-Scale WSN for Water Environment Monitoring with Application to Three Gorges Reservoir Area

**DOI:** 10.1155/2014/802915

**Published:** 2014-03-05

**Authors:** Yuanchang Zhong, Lin Cheng, Liang Zhang, Yongduan Song, Hamid Reza Karimi

**Affiliations:** ^1^College of Communication Engineering, Chongqing University, Chongqing 400044, China; ^2^School of Automation, Chongqing University, Chongqing 400044, China; ^3^Department of Engineering, Faculty of Engineering and Science, University of Agder, N-4898 Grimstad, Norway

## Abstract

The typical application backgrounds of large-scale WSN (wireless sensor networks) for the water environment monitoring in the Three Gorges Reservoir are large coverage area and wide distribution. To maximally prolong lifetime of large-scale WSN, a new energy-saving routing algorithm has been proposed, using the method of maximum energy-welfare optimization clustering. Firstly, temporary clusters are formed based on two main parameters, the remaining energy of nodes and the distance between a node and the base station. Secondly, the algorithm adjusts cluster heads and optimizes the clustering according to the maximum energy-welfare of the cluster by the cluster head shifting mechanism. Finally, in order to save node energy efficiently, cluster heads transmit data to the base station in single-hop and multihop way. Theoretical analysis and simulation results show that the proposed algorithm is feasible and advanced. It can efficiently save the node energy, balance the energy dissipation of all nodes, and prolong the network lifetime.

## 1. Introduction

In recent years, the WSN technology has attracted extensive attention of the academia, industry and, government. It became one of the most competitive technologies among a lot of fields, such as, national defense and military, environment monitoring and forecasting, healthcare, smart home, building structure monitoring, complex mechanical control, urban transportation, space exploration, management of large workshop and warehouse, and safe monitoring of airport and large industrial parks [1, 2]. However, these sensor nodes in WSN are usually powered by batteries; the energy of nodes is limited and it is difficult to replace or recharge the batteries because of the wild environment and the huge number of nodes. The limited energy of nodes seriously affects the lifetime of WSN, which has constrained the large-scale application of WSN. Hence, it is important to improve the energy efficiency and prolong the network lifetime under the premise of limited energy, which is the key technical problems of WSN needing to be solved [3].

Designing an energy-efficient routing algorithm is the best way to prolong network lifetime of WSN and improve the quality of information transmission [4, 5]. Currently, a variety of routing protocols have been proposed to save energy. Existing routing protocols are generally divided into two categories: flat routing and hierarchical routing [6]. Flat routing is easy to implement, no additional cost of topology maintenance and packet routing, but it is not proper to wireless sensor networks. Hierarchical routing, also known as clustering routing, such as low-power Adaptive Clustering Hierarchy Protocol LEACH [7] and PEGASIS [8] protocols, has proposed the methods using cluster heads to form the clusters. Researches show that the hierarchical routing protocol is better than the planar routing protocol in the aspects of adaptability and energy efficiency. Thus hierarchical routing protocol received extensive attention [9, 10].

The application of wireless sensor networks for water environment monitoring in the Three Gorges Reservoir area is a typical case of large-scale wireless sensor networks research. And it has the typical characteristics of zonal distribution and large coverage area. Our research is based on the achievements we already obtained and deeply study on the existing routing algorithms, such as, LEACH and PARPEW [11] algorithm, and the focus is on the energy saving routing algorithm for large-scale WSN. So an improved energy-saving routing algorithm based on maximum energy-welfare optimization clustering has been proposed. In the simulation experiment, a rectangular area with greater aspect ratio is used to approach the actual banding distribution. Simulation results show that the performance of the proposed algorithm is superior to the other two algorithms'.

## 2. The Network Model of Large-Scale Wireless Sensor Networks

The Three Gorges Project is the world-famous water conservancy project, which brings the enormous economic and social benefits, while also triggering ecological environmental safety issues. Because that the self-purification capacity of static water is much lower than flowing water, the Three Gorges Reservoir water environment was gradually deteriorating and the water environment security became the focus of attention at home and abroad [12]. Currently, the environmental monitoring stations have been established in the Three Gorges Reservoir area to undertake a very important water environment monitoring tasks. However, the existing monitoring equipment, monitoring methods and means cannot meet the requirements, so it is urgent to strengthen the reservoir water environment monitoring capacity and improve the level of monitoring. According to this situation, the solution of building the Three Gorges Reservoir water environment monitoring system with WSN has been proposed [13].

**Figure 1:**
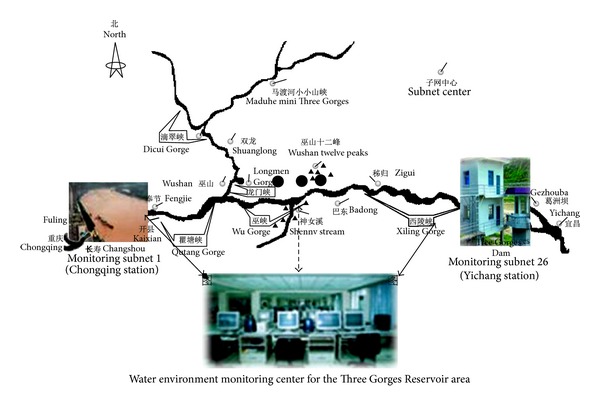
Distribution of the Three Gorges Reservoir and structure model of WSN water quality monitoring system.

Due to the large area and wide distribution of the Three Gorges Reservoir, which involves 26 counties (of which 22 are in Chongqing Municipality's jurisdiction) and some less accessible places, it shows a meandering tree structure [14]. Therefore, the distribution and networking method should be based on its unique serpentine tree structure. The distribution of the Three Gorges Reservoir and the structure model of the WSN water quality supervision system are shown in Figure 1.

Figure 1 shows that in such a large-scale wireless sensor network, nodes are far away from each other, and energy consumption by sending messages among nodes is very large, especially in some remote and inaccessible areas. So it is unrealistic to replenish their nodes energy frequently. Therefore, it is particularly important to improve energy efficiency and balance energy consumption of nodes in the wireless sensor networks. For these characteristics of the network, a new energy-saving routing algorithm has been proposed to solve this problem.

## 3. Related Researches

LEACH is the first to be proposed as a hierarchical routing algorithm, which combines the MAC protocol and the cluster-based energy efficient routing algorithm and performs well in extending the lifetime of the network. Dividing the network running into many rounds is its main idea, and in each round there are two steps: clusters establishment and stable transmission of data. During the establishment of clusters, each node generates a random value between 0 and 1. And if it is less than the threshold value *T*(*n*), expressed as (1), it will be elected as the cluster head:

(1) $$\begin{array}{c} T\left(n\right)=\{\begin{array}{cc} \frac{p}{1-p\left(r\mathrm{mod}\left(1/p\right)\right)}, & n\in G\\ 0 & n\notin G, \end{array} \end{array}$$

where, *p* is the percentage of the cluster heads occupied in the total nodes, *r* is the current round number, and *G* is the set of the nodes which have not been selected as cluster heads in the last 1/*p* rounds.

In the LEACH algorithm, the random rotation mechanism of cluster heads was used to balance the energy load to each node, so that the network lifetime has been prolonged. Compared with the normal planar multihop routing algorithm and static hierarchical algorithm, LEACH algorithm is outstanding, and the network lifetime can be prolonged by 15%. However, there are also some shortcomings, such as: (1) the remaining energy of nodes was not considered into the cluster heads selection; (2) uneven distribution of cluster heads and cluster sizes resulted from the random cluster heads selection mechanism, which causes the decline in the balance of network load; (3) when the network size is large, single-hop data transmission will lead to those cluster heads death, which are far away from the base station, so that the lifetime of the whole network will be affected.

Therefore, a lot of improved algorithms based on LEACH have been proposed to improve the network performance recently. In the LEACH-C algorithm [15], which was proposed by Heinzelman and so forth, the centralized control method was used and the base station obtained the cluster head by running a simulated annealing, so that the problem of uneven distribution of cluster heads in LEACH was solved. But it is not completely self-directed and is not good in expansibility with the centralized control method. To improve the quality of clustering, the distribution density, remaining energy of nodes, and other factors were taken into consideration in [16, 17]. But collecting information about neighbor nodes in the whole network increased the communication overhead and latency. In [18], the time interval was used to cluster instead of random number and threshold. But the distribution of the cluster head nodes has been ignored. A clustering algorithm based on energy optimization has been proposed in [19], which protected low energy nodes and reduced overhead of clustering by the competition parameters of cluster head, so that the network lifetime was prolonged. In [20], firstly, clusters were formed according to the optimization of the number of clusters, and then the data routing among clusters were completed by searching the shortest path based on the Kruskual algorithm, so that the energy efficiency of nodes has been improved. But the balance of the energy consumption of network was not mentioned.

In the social economics, the welfare reflects the equilibrium relationship between fairness and efficiency of the social crowd income [21]. In [21], a new metric for WSN is proposed, which combines energy balancing and energy efficiency, that is, EW (energy-welfare). The main idea of the PARPEW algorithm is to optimize clustering according to the predicted energy consumption of nodes and energy-welfare of clustering. So, good results such as extending network lifetime and balancing energy consumption have been achieved. Besides, it is simple to compute the energy welfare only through the local routing information. However, the shortcoming is that in the cluster heads adjustment, only the energy welfare was taken into consideration, while the effect of the remaining energy of nodes was ignored. Therefore, the situation that those nodes with low remaining energy were elected as the cluster head also existed. In addition, communication using single-hop will decrease the energy efficiency and is not conducive to network expansion.

## 4. The Improved Algorithm

### 4.1. The Network Model

Assume that the wireless network has the following properties.

1. The position of the nodes will not be changed once deployed. The base station is located outside the area and full of energy.
2. All nodes are energy limited and of the same structure and initial energy.
3. All nodes have a unique ID.
4. The transmit power can be adjusted according to the distance.
5. All nodes can sense their remaining energy and compute the distance to the emission source according to the received signal strength.

### 4.2. The Energy Model

**Figure 2:**
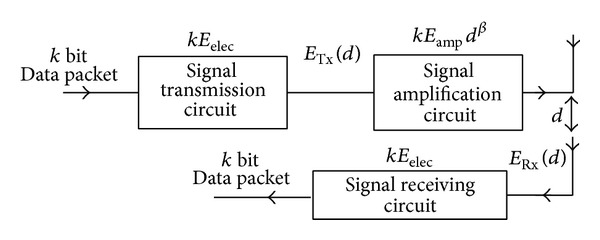
The first-order energy model.

The energy model which is the same with it in [7] was used in this paper, as shown in Figure 2.

Figure 2 shows the data packet transmission between two nodes, in which the distance is *d* and the size of the packet is *k* bit. So the transmitter energy consumption is expressed as:

(2) $$\begin{array}{c} E_{\text{Tx}}\left(k,d\right)=\{\begin{array}{cc} kE_{\text{elec}}+k\varepsilon_{fs}d^{2}, & d<d_{0}\\ kE_{\text{elec}}+k\varepsilon_{\text{amp}}d^{4}, & d>d_{0}. \end{array} \end{array}$$

The energy consumption of the receiving terminal is shown in as:

(3) $$\begin{array}{c} E_{\text{Rx}}\left(k\right)=kE_{\text{elec}}. \end{array}$$

In (2) and (3), the energy consumption of transmitting and receiving 1 bit data is denoted as *E*_elec_. *ε*_*fs*_, *ε*_amp_ are the energy consumption coefficients of different channel propagation model. *d*_0_ is the threshold value denoted as $d_{0}=\sqrt{\varepsilon_{fs}/\varepsilon_{\text{amp}}}$, which is used to distinguish the free-space path loss model from the multipath fading model.

The energy consumption for integration of *l* data packets of *k* bit is expressed as:

(4) $$\begin{array}{c} E_{\text{DA}}\left(k\right)=l\times k\times E_{\text{DA}}, \end{array}$$

where, *E*_DA_ is the energy consumption for integration of a data of 1 bit.

### 4.3. Algorithm Description

In the initial stages of networking, the TDMA time slot of the whole nodes was broadcasted by the base station at a time. Each node broadcasted its message to the whole nodes according to the TDMA time slot and then calculated the distance from the emission source according to the received signal strength, where the distance is denoted as *d*(*i*, *j*),  *i*,  *j* = 1,2,…, *n*, and *n* is the number of the nodes in the network. And to provide convenience for next clustering optimization and routing, the distance was stored in the memory of the node. This step only runs once at the initial stage of networking, and the following operation model was the same with the LEACH algorithm.

#### 4.3.1. Temporary Clustering

To reduce the energy consumption of nodes, it is better to select the nodes with more remaining energy near the base station. So, in this paper, the two factors were all taken into consideration when selecting the cluster heads. The improved threshold *T*(*n*) is expressed as:

(5) $$\begin{array}{c} T\left(n\right)=\{\begin{array}{cc} \frac{p}{1-p\left(r\mathrm{mod}\left(1/p\right)\right)}\left[E\left(n\right)+D\left(n\right)\right], & n\in G\\ 0 & n\notin G, \end{array} \end{array}$$

where *E*(*n*) is the energy factor, denoted as *E*(*n*) = *E*_res_(*n*)/*E*_init_; *E*_res_(*n*), *E*_init_ are the remaining energy and initial energy of the node *n*, respectively. *D*(*n*) is the distance factor, expressed as *D*(*n*) = 1 − [*d*(*n*, BS)/*d*_max⁡_], *d*(*n*, BS) is the distance between node *n* and base station, and *d*_max⁡_ is the maximum of the distance from nodes to base station.

After the election of the temporary cluster head, the normal nodes sent application (including their own ID and the remaining energy of node) to those cluster heads, which costed their least communication energy. Thus, the temporary clustering was completed.

#### 4.3.2. Cluster Heads Adjustment Based on Energy Welfare Maximization and Clustering Optimization

*(1)  Energy Welfare.* Assume that the *r*th round of the cluster *C* has *N* nodes, and the remaining energy of the *i*th node is denoted as *E*_res_^*r*^(*i*). According to the network model, the remaining energy after transmission of the node *i* can be predicted, which is expressed as ${\bar{E}}_{\text{AT}}^{r}$, shown as follows:

(6) $$\begin{array}{c} {\bar{E}}_{\text{AT}}^{r}=\frac{1}{N}\sum_{i\in N}E_{\text{AT}}^{r}\left(i\right). \end{array}$$

Thus, the energy welfare of cluster *C* can be obtained [21], as expressed in the following equations:

(7) $$\begin{array}{c} EW_{C}^{r}\left(\varepsilon \right)={\bar{E}}_{\text{AT}}^{r}\times EE_{C}^{r}\left(\varepsilon \right),\\ EE_{C}^{r}\left(\varepsilon \right)=1-I_{C}^{r}\left(\varepsilon \right),\\ I_{C}^{r}\left(\varepsilon \right)=1-{\left[\frac{1}{N}{\sum_{i\in N}\left(\frac{E_{\text{AT}}^{r}\left(i\right)}{{\bar{E}}_{\text{AT}}^{r}}\right)}^{1-\varepsilon }\right]}^{1/\left(1-\varepsilon \right)}, \end{array}$$

where,  *EE*_*C*_^*r*^(*ε*) is the balance index of the energy consumption of node, and the greater the value, the more balanced the energy consumption. *ε* is the parameter representing the degree of hating inequality, and the greater the value of *ε*, the more disagreeable of inequality. So, the energy consumption of nodes with low power attracts more attention, and the typical value for *ε*  is 1.5 [22].

**Figure 3:**
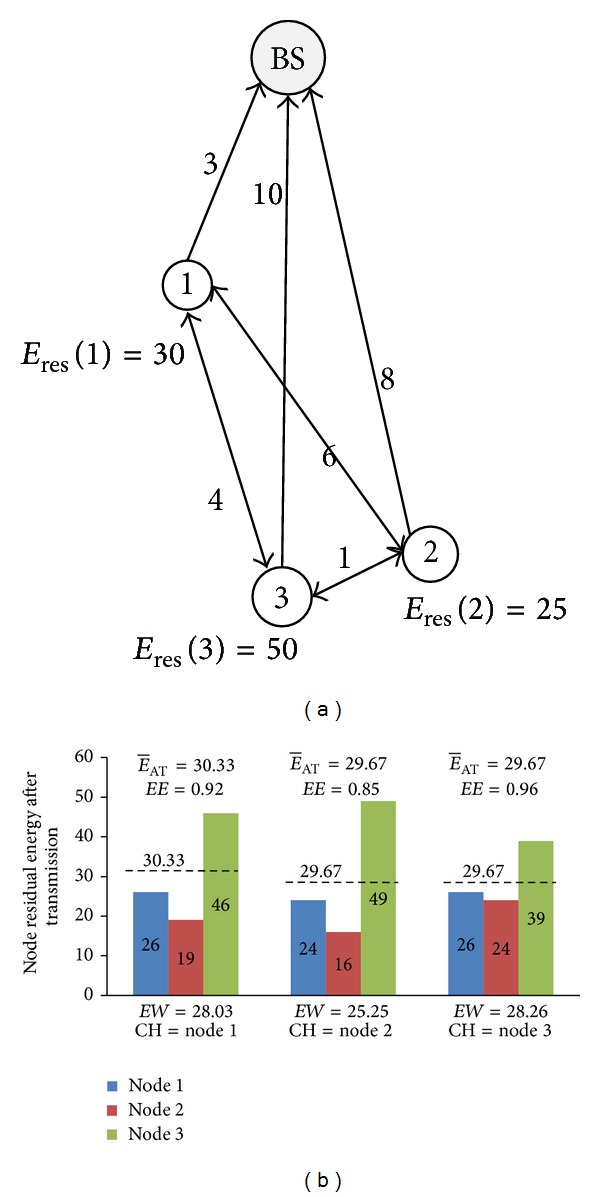
The instance of the energy welfare. (a) Energy consumption relationship between nodes. (b) Comparison between remaining energy of node and clustering welfare.

The energy efficiency and the property of energy balance were both considered into the energy welfare. Therefore, increasing energy welfare can save node energy and balance the energy consumption of the network. The energy welfare instance is shown in Figure 3.

As is shown in Figure 3(a), there are three nodes in the cluster, and the energy required by the data transmission among nodes is given, and *E*_res_(*i*) is the remaining energy of the node *i*. To simplify the calculation, the energy consumed by cluster head for receiving data was set to be 1. The comparison between remaining energy after transmission and energy welfare of clustering of the three kinds of network models is shown in Figure 3(b). As can be seen in the figure, when the node 1 was selected as cluster head (CH), the energy efficiency is maximal, and the mean of remaining energy is 30.33 J. Considering the energy consumption balance index, when the node 3 was selected as cluster head, the energy welfare of clustering reached the maximum, and the energy efficiency and the property of energy consumption are the best. Therefore, to elect node 3 as cluster head is the most reasonable.

(*2) Cluster Head Adjustment and Process of Clustering Optimization*. Before cluster head adjustment, the candidate nodes for cluster head should be limited firstly. Assuming that the set of the candidate nodes for cluster head of the cluster *C* is *Q*, so

(8) $$\begin{array}{c} Q=\left\{i\;|\;i\in C,\;\;E_{\text{res}}^{r}\left(i\right)>{\bar{E}}^{r}\right\}, \end{array}$$

where, *E*_res_^*r*^(*i*) is the remaining energy of node *i* in the *r*th round, and ${\bar{E}}^{r}$ is the mean value of the energy remaining of the nodes as follows:

(9) $$\begin{array}{c} {\bar{E}}^{r}=\frac{1}{N}\sum_{i\in N}E_{\text{res}}^{r}\left(i\right). \end{array}$$

The processes of clustering optimization are shown as the following.

1. Calculate the energy array according to the network model and the distance array *d*(*i*, *j*), and then determine the set *Q* of candidate nodes for cluster head of cluster *C*.
2. If the set *Q* is empty, the temporary cluster head will be selected as the real cluster head (RCH) directly. Otherwise, the energy welfare of cluster *C* is *EW*_*C*_^*r*^|_(RCH=*i*)_,  *i* ∈ *Q* according to equations (6)-(7) using each node in the set *Q* when the node *i*  (*i* ∈ *Q*) is selected as RCH.
3. Determine RCH according to the max energy-welfare of cluster *C*, that is max⁡_*k*∈*Q*_⁡*EW*_*C*_^*r*^, and the node *k* is RCH.
4. After RCH is determined, the temporary cluster head broadcasts the TDMA time slot and control packets (including the ID of the temporary cluster head and RCH, and the remaining energy) of the cluster member nodes. Other nodes in the cluster become the normal nodes according to the control packets and send data to RCH according to the distributed TDMA time slot. At last the cluster head adjustment and clustering optimization are finished.

#### 4.3.3. The Data Routing among Clusters

The transmission of data routing among nodes combines single-hop with multihop. If the distance from cluster head to base station is less than *d*_0_, the cluster head communicates with base station directly. Otherwise, a relay will be chosen from the neighbor cluster heads to transmit data until the data reach the base station.

Firstly, the set of neighbor cluster heads of cluster *C*_*i*_ (*i* = 1, 2 … *K*,  *K* is the number of the cluster heads) is denoted as *S* which should meet the following condition:

(10) $$\begin{array}{c} S=\left\{C_{j}\;|\;\alpha <\frac{d\left(C_{j},\text{BS}\right)}{d\left(C_{i},\text{BS}\right)}<1,\;\;d\left(C_{i},C_{j}\right)<d\left(C_{j},\text{BS}\right)\right\}, \end{array}$$

where *d*(*C*_*i*_, *C*_*j*_) is the distance from cluster head *C*_*i*_ to cluster head *C*_*j*_, and *d*(*C*_*i*_,  BS) is the distance from cluster head *C*_*i*_ to base station. *α* is the forward cost factor, and 0.8 < *α* < 1. Choosing a proper *α* can avoid the problem that those cluster heads of the same distance away from base station consume too much energy for transmitting data.

Then, using the greedy algorithm the cluster head *C*_*i*_ selects a relay which has the maximal weight function from the set *S* to transmit data. Considering the factors, such as, remaining energy of the node, the path costs, and the angle deviation between node and base station [23], the weight function is defined as follow:

(11) $$\begin{array}{c} W=u\frac{E_{\text{res}}\left(j\right)}{E_{\text{res}}\left(i\right)}+v\cos\theta +w\frac{d{\left(C_{i},\text{BS}\right)}^{2}}{d{\left(C_{i},C_{j}\right)}^{2}+d{\left(C_{j},\text{BS}\right)}^{2}},\\ \cos\theta =\frac{d{\left(C_{i},\text{BS}\right)}^{2}+d{\left(C_{i},C_{j}\right)}^{2}-d{\left(C_{i},\text{BS}\right)}^{2}}{2d\left(C_{i},\text{BS}\right)\times d\left(C_{i},C_{j}\right)}, \end{array}$$

where *E*_res_(*j*) is the remaining energy of the cluster head *C*_*j*_, *θ* is the angle deviation between node and base station, *μ*, *ν*, *ω* are the weighting coefficient, and *μ* + *ν* + *ω* = 1. If the set *S* is empty, the cluster head *C*_*i*_ sends data to the base station directly. Otherwise, the cluster head *C*_*i*_ sends data to the relay. When all of the cluster heads find the relays, the data routing among clusters is completed.

## 5. Simulation and Analysis

**Figure 4:**
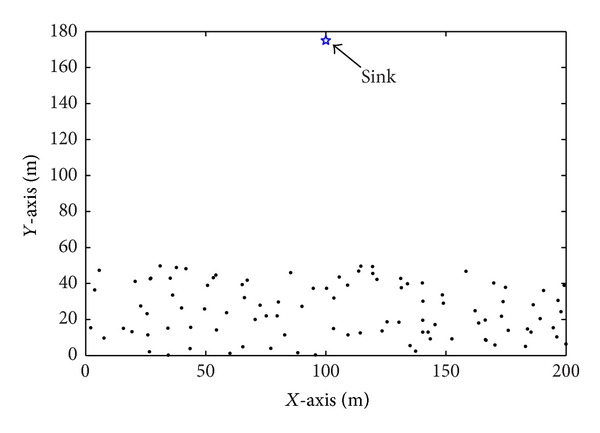
100-node random network.

The performance evaluation of our proposed algorithm has been carried by using MATLAB simulation. We also compared the proposed algorithm with the LEACH algorithm and PARPEW algorithm. Taking into account the typical characteristics of zonal distribution of the Three Gorges Reservoir for water environment monitoring, in the simulation experiment, we used a rectangular area to approach the banding distribution, which has greater aspect ratio. Network simulation scenario is shown in Figure 4.

100 sensor nodes were deployed in a 200 m × 50 m rectangular area, and the base station was just above the area with the coordinates (100 m, 175 m). In the simulation, the forward price factor *α* = 0.9, the weighting coefficients *μ* = 1/2,  *ν* = 1/6,  *ω* = 1/3. Other simulation parameters of the network are shown in Table 1.

First, we compared the network performance among three algorithms, including the number of surviving nodes, nodes' energy consumption, and energy balance of network. Then, we analyzed the performance of our proposed algorithm in large-scale wireless sensor networks.

### 5.1. The Simulation of Lifecycle and Energy Consumption

**Figure 5:**
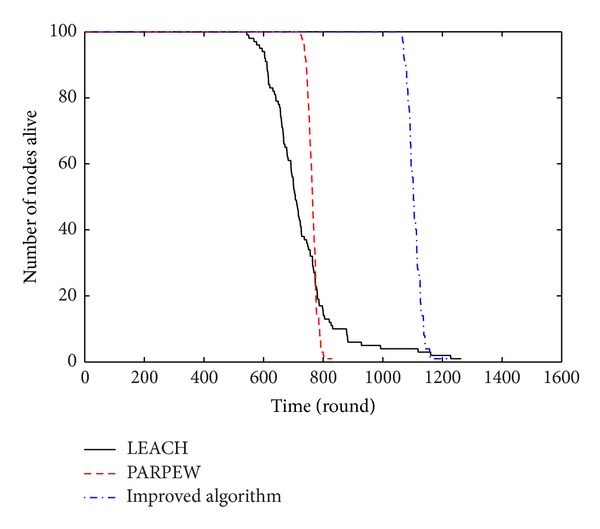
The relationship between the number of the survival nodes and the rounds.

Network lifetime is one of the most important criteria for evaluating the quality of routing protocol.  Figure 5 shows the corresponding changes of the survival nodes' number during simulation. As can be seen from the figure, the network lifetime (defined as the number of rounds when the first node died) was obviously improved by the proposed algorithm in this paper with respect to LEACH and PARPEW algorithms. Since the proposed algorithm in this paper is based on the improved max energy-welfare method to adjust cluster head and optimize the clustering, the distribution of the cluster heads is more uniform and reasonable, and the energy consumption is efficiently balanced. Besides, cluster heads transmit data to the base station in single-hop and multi-hop way. It improves the energy efficiency of cluster heads and balances the energy consumption of cluster heads in different place.

**Figure 6:**
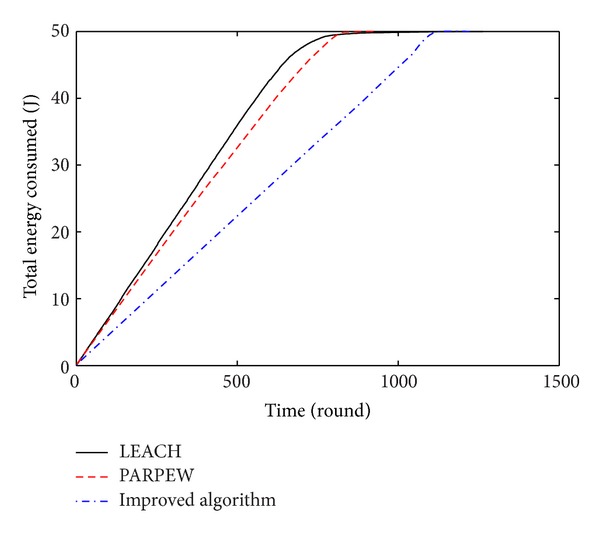
The changing curves of the energy consumption of network.

The comparison curve of the total energy consumption of the network and the simulation time among the three algorithms is shown in Figure 6. The smaller slope of the curve indicates that the network has a slower rate of energy consumption and longer lifetime. In Figure 6, the slope of the curve of the proposed algorithm in this paper is minimal, and the total energy consumption of the network in any time is lower than that of the LEACH and PARPEW algorithms. So the energy consumption of the network has been reduced successfully.

To eliminate the effect of the chance factor, a number of simulations have been done to obtain the statistical average. The statistical average of lifetime and the total energy consumption at 500th round using different algorithms are shown in Table 2.

As can be seen from Table 2, compared with other two algorithms, the proposed algorithms can efficiently extend the lifetime of the network and reduce network energy consumption. The detailed comparative results are shown in Table 3.

### 5.2. The Simulation of Energy Balance

**Figure 7:**
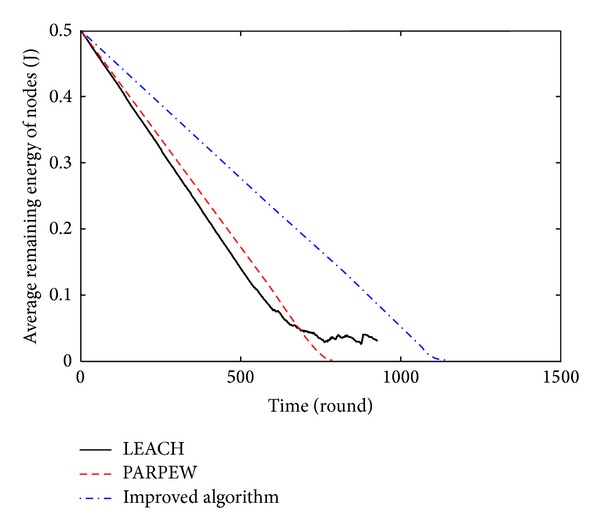
Comparison of the remaining energy average.

**Figure 8:**
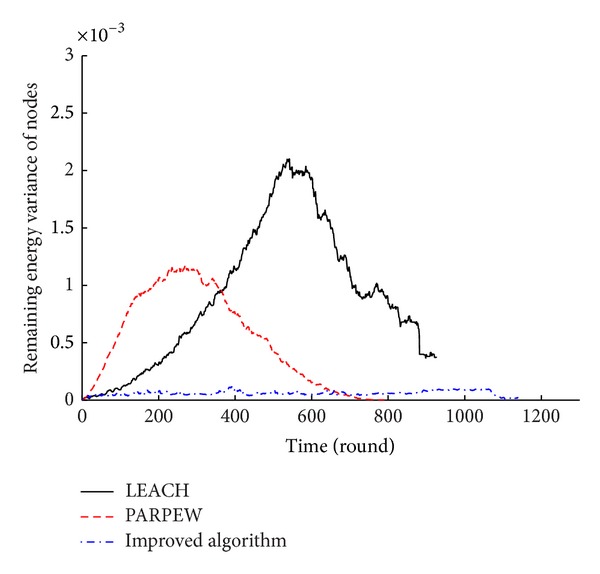
Comparison of the remaining energy variance.

In this paper, the remaining energy mean and variance of nodes at a certain time were used to measure and further analyze the energy balance of the proposed algorithm. At time *t*, the greater the mean and the less the variance, the better the energy balance of the network. The comparison of the energy balance among the different algorithms is shown in Figures 7 and 8.

Figures 7 and 8 show that the average remaining energy of nodes in the network of the proposed algorithm in this paper has been greater than the mean energy of the other two algorithms, and the variance of the residual energy is much lower than the other two algorithms. There is no much change. Therefore, the proposed algorithm in this paper is effective in saving nodes' energy and balancing the network energy consumption. Its performance of energy balance is optimal.

### 5.3. The Simulation of Adaptability in Large-Scale WSN

Another important feature of the Three Gorges Reservoir water environment monitoring network is large coverage area and wide distribution. To further illustrate our proposed algorithm's good performance in large-scale wireless sensor network applications, we assumed three-network simulation environments as shown in Table 4. And we compared the network lifetime of three algorithms under the same conditions of the other parameters.

**Figure 9:**
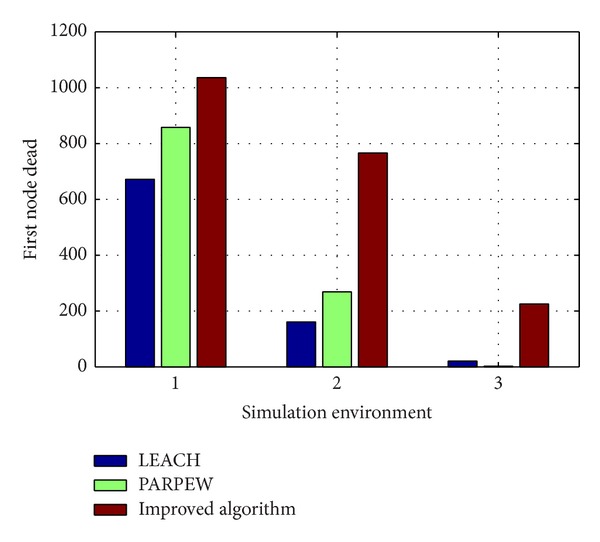
Impact of the simulation environment on the FND of the compared algorithms.

Figure 9 shows the comparison of the network lifetime of three algorithms under different environment conditions. As can be seen from the figure, in the three simulation environments, network lifetime of the proposed algorithm is superior to the other two algorithms'. And with the increasing of network size, the advantages of the proposed algorithm are more obvious in performance. This is mainly because that the algorithm uses the cluster heads optimization strategies and improved data transmission scheme to fully save and balance the energy consumption of nodes. Therefore, for the feature of large-scale water environment monitoring network application in Three Gorges Reservoir, our proposed algorithm has obvious advantages.

## 6. Conclusions

An improved energy saving routing algorithm based on maximum energy-welfare optimization clustering has been proposed. There are three aspects of the major improvements: firstly, remaining energy and the distance from base station of nodes were both considered when selecting cluster heads. So it is more reasonable. Secondly, the cluster heads adjustment and clustering optimization based on improved maximum energy-welfare made the distribution of cluster heads more uniform, and the energy consumption of network has been balanced efficiently. Thirdly, the factors such as path costs, remaining energy, and angle deviation between node and base station were taken into consideration in the clustering routing construction, so that the energy efficiency of nodes has been increased. Simulation results show that compared with LEACH and PARPEW algorithms, the proposed algorithm in this paper can efficiently save energy of each node, balance the energy consumption, and prolong the lifetime of the network. It is suitable to the Three Gorges Reservoir water environment monitoring network with zonal structure. With the increasing size of network, this effect will be more obvious.

## Figures and Tables

**Table 1:** The parameters of the network in simulations.

Parameters	Values
Initial energy of nodes *E*_init_	0.5 J
Amplification coefficient of the free space model *E*_fs_	10 pJ·m^2^/b
Amplification coefficient of the multipath transmission model *E*_amp_	0.0013 pJ·m^2^/b
Data fusion rate *E*_DA_	5 nJ/b
Circuit loss *E*_elec_	50 nJ/b
Clustering probability of nodes *p*	0.05
Data packet length	4000 b
Control packet length	80 b

**Table 2:** The statistical average of lifetime and energy consumption of network.

Algorithms	LEACH	PARPEW	The proposed
FND	542	713	1049
Total energy consumption at 500th round (J)	35.95	32.66	22.32

**Table 3:** The percentage of comparison of lifetime and energy consumption of network.

Comparison parameters	Comparison with LEACH	Comparison with PARPEW
FND (increased percentage)	93.5	47.1
Total energy consumption at 500th round (reduced percentage)	37.9	31.7

**Table 4:** Three kinds of simulation environments.

Simulation environment	Area (m^2^)	Base station Location (m)	Number of nodes
1	100 × 100	(100, 175)	100
2	200 × 200	(100, 175)	400
3	400 × 400	(100, 175)	800
